# Comparative salivary proteomics analysis of children with and without dental caries using the iTRAQ/MRM approach

**DOI:** 10.1186/s12967-018-1388-8

**Published:** 2018-01-19

**Authors:** Kun Wang, Yufei Wang, Xiuqing Wang, Qian Ren, Sili Han, Longjiang Ding, Zhongcheng Li, Xuedong Zhou, Wei Li, Linglin Zhang

**Affiliations:** 1 State Key Laboratory of Oral Diseases, West China Hospital of Stomatology, Sichuan University, No. 14, Section 3 of Renmin South Road, Chengdu, Sichuan China; 2 National Clinical Research Center for Oral Diseases, West China Hospital of Stomatology, Sichuan University, No. 14, Section 3 of Renmin South Road, Chengdu, Sichuan China; 3 Department of Cariology and Endodontics West China Hospital of Stomatology, Sichuan University, No. 14, Section 3 of Renmin South Road, Chengdu, Sichuan China

**Keywords:** Dental caries, Children, Salivary proteome, iTRAQ, MRM

## Abstract

**Background:**

Dental caries is a major worldwide oral disease afflicting a large proportion of children. As an important host factor of caries susceptibility, saliva plays a significant role in the occurrence and development of caries. The aim of the present study was to characterize the healthy and cariogenic salivary proteome and determine the changes in salivary protein expression of children with varying degrees of active caries, also to establish salivary proteome profiles with a potential therapeutic use against dental caries.

**Methods:**

In this study, unstimulated saliva samples were collected from 30 children (age 10–12 years) with no dental caries (NDC, n = 10), low dental caries (LDC, n = 10), and high dental caries (HDC, n = 10). Salivary proteins were extracted, reduced, alkylated, trypsin digested and labeled with isobaric tags for relative and absolute quantitation, and then they were analyzed with GO annotation, biological pathway analysis, hierarchical clustering analysis, and protein–protein interaction analysis. Targeted verifications were then performed using multiple reaction monitoring mass spectrometry.

**Results:**

A total of 244 differentially expressed proteins annotated with GO annotation in biological processes, cellular component and molecular function were identified in comparisons among children with varying degrees of active caries. A number of caries-related proteins as well as pathways were identified in this study. As compared with caries-free children, the most significantly enriched pathways involved by the up-regulated proteins in LDC and HDC were the ubiquitin mediated proteolysis pathway and African trypanosomiasis pathway, respectively. Subsequently, we selected 53 target proteins with differential expression in different comparisons, including mucin 7, mucin 5B, histatin 1, cystatin S and cystatin SN, basic salivary proline rich protein 2, for further verification using MRM assays. Protein–protein interaction analysis of these proteins revealed complex protein interaction networks, indicating synergistic action of salivary proteins in caries resistance or cariogenicity.

**Conclusions:**

Overall, our results afford new insight into the salivary proteome of children with dental caries. These findings might have bright prospect in future in developing novel biomimetic peptides with preventive and therapeutic benefits for childhood caries.

**Electronic supplementary material:**

The online version of this article (10.1186/s12967-018-1388-8) contains supplementary material, which is available to authorized users.

## Background

Paediatric dental caries is one of the most common chronic infectious diseases in childhood of great concern to parents and dentists, affecting up to 60% of schoolchildren in China, and remains a major problem in many countries [1]. Cariogenic bacteria, cariogenic diets, susceptible host and affected time together contribute to the occurrence of dental caries. Caries causes lesions and cavities on tooth surfaces, leading to decay and even loss of tooth structure. The destruction can progress in a rapid speed if left untreated, resulting in pain and infection. Therefore, early diagnosis and prevention are of particular clinical significance [2, 3]. Current studies seek to identify the risk factors for caries as well as to study oral defense functions in protecting against and preventing the development of this disease [4]. The known factors influencing dental caries in children include: immature immune systems, cariogenic microorganisms, characteristics of saliva, and oral hygiene care in childhood [5]. As one of the most important host-associated factors in the etiology of caries, saliva contacts closely with teeth, and the constituents of this biological fluid play an essential role in the occurrence and progression of dental caries [6, 7].

Human whole saliva is primarily composed of water and originated mainly from three major salivary glands—parotid, submandibular and sublingual glands [8], involving in maintenance of oral homeostasis [9]. As the principal component of saliva, salivary proteins, although accounting for only a small proportion of saliva, play various important roles to keep the integrity of teeth depending on their ability to inhibit the growth of cariogenic bacteria or to modulate the demineralization/remineralization balance, such as lactoferrin, lysozyme, proline-rich proteins and statherin [10]. Besides of these anti-cariogenic factors in saliva, there are many proteins taking part in the cariogenic progress through promoting the proliferation and tooth colonization of bacteria [7]. For example, common salivary protein-1 was suggested to be able to enhance the binding of *Streptococcus mutans* to the hydroxyapatite surface, indicating its potential influence on the initial colonization of pathogenic bacterium onto the tooth surface [11]. In addition, elevated levels of salivary matrix metalloproteinase (MMP)-8 were found in patients with dental caries relative to healthy individuals, which was supposed to initiate the collagen degradation in caries process in dentin [12]. Therefore, understanding the role of proteins in cariogenic saliva will be of great importance for both the assessment of caries susceptibility and caries prevention.

Mass spectrometry (MS)-based proteomics is a large-scale, high-throughput, systematic study, allowing for the comprehensive characterization of salivary proteins, even with a limited amount of samples [13]. Easy and non-invasive collection of saliva made it interesting to be used for the assessment of a variety of oral diseases applying these techniques, such as Sjögren’s syndrome, oral squamous cell carcinoma and periodontitis [14–16]. Also, salivary protein profiles of dental caries have been investigated in the last decade, but the results lacking of validation for candidate proteins still remain controversial. A high degree of similarity in the general composition of salivary proteins was shown between children with and without dental caries in a previous study [17]. On the contrary, another study identified significant differences in salivary protein expression profiles between children with severe childhood caries and caries-free children [18]. More recently, overexpression of salivary complement system and inflammatory markers were demonstrated in caries patients compared to healthy controls [19]. Therefore, the relationship between dental caries and human salivary proteins is yet to be well defined, and the biomarker information remains unclear.

Isobaric tags for relative and absolute quantitation (iTRAQ) is one of the new techniques used in modern proteomics that couples stable isotopes labeling and tandem mass spectrometry to permit comparative quantification with good precision. Multiple reaction monitoring (MRM) is a powerful tool for targeted proteomics and is an emerging field of proteomics with high reproducibility across complex samples. To date, several studies have demonstrated the feasibility of using targeted MRM-MS for quantitative proteomic analyses, which could realize highly multiplex, precise, specific and standardized proteomic quantification [20, 21]. Also, iTRAQ discovery combined with subsequent MRM conformation has recently been adopted for disease biomarker quantification studies [22, 23], but it has rarely been used for the salivary proteomic analysis of childhood caries.

In the current study, for the first time, we applied iTRAQ as a discovery method, followed by a verification (MRM) to perform a comparative saliva proteomics analysis for identifying key candidate proteins with diagnostic or protective value for childhood caries. Our results will serve to better understand the roles of salivary proteins involved in the onset and progression of childhood caries as well as their potential impact on clinical practice for anti-caries.

## Methods

### Subjects recruitment and samples collection

All human saliva samples were collected from children aged between 10 and 12 years attending the first primary school of Emei, Sichuan, China. Dental examinations were performed by 5 professional dentists who had previously trained for the evaluation and sampling procedures according to the criteria defined by the National Institute of Dental and Craniofacial Research (NIDCR; USA) for caries diagnosis and recording [24]. The DMFT/dmft index measures the number of decayed, missing and filled teeth in epidemiologic surveys of dental caries [25]. All Children sharing a relatively homogeneous school living environment were divided into three groups according to the severity of dental caries: no dental caries (NDC) group (n = 10, DMFT = 0), low dental caries (LDC) group (n = 10, DMFT/dmft ranging from 1 to 4), and high dental caries (HDC) group (n = 10, DMFT/dmft ranging from 5 to 10). All subjects were willing to consent to the clinical examination and saliva sampling. Those with other detectable oral disease or severe systemic disorders, and those who had received antibiotic therapy within 1 month were excluded. This study was approved by the Ethics Committee of West China Hospital of Stomatology, Sichuan University. Informed consent was obtained from the guardians of all children.

According to the standard techniques described by Navazesh [26], about 3 ml spontaneous, whole unstimulated saliva was collected in a sterile enzyme-free conical tube from each subjects between 9:00 and 11:00 a.m. Subjects were instructed to refrain from drinking and eating for at least 2 h before sampling [27]. After collection, all samples were kept on ice and immediately transported to laboratory. The saliva proteome samples were centrifuged at 12,000 rpm for 5 min at 4 °C, and the supernatants were treated with a protease inhibitor mixture (2 μl/ml; Sigma-Aldrich, St Louis, MO, USA), and then divided into smaller volumes and stored at − 80 °C until further use. To avoid issues with protein degradation, we did not reuse thawed saliva samples [28].

### Proteins extraction and qualification

Salivary protein was extracted by acetone precipitation method as described previously [29] with some modification. Whole saliva was mixed with solubilization buffer (8 M urea, 30 mM HEPES, 1 mM PMSF, 2 mM EDTA, 10 mM DTT). The mixture was sonicated with 2 cycles of 5 s, and then was centrifuged at 20,000×*g* 30 min at 4 °C. The pellet was discarded and the supernatant was saved for analysis. Protein quantification was performed using Bradford assay (Bio-Rad, Hercules, California, USA) with bovine serum albumin (BSA) as standard.

### iTRAQ analysis

#### iTRAQ labeling of tryptic peptides derived from salivary proteins

Reduction, alkylation and trypsin digestion of salivary proteins were performed as described previously [30] with some modification. 100 μg treated salivary proteins from each different group were digested using trypsin (Promega, Madison, USA) at 37 °C for 16–18 h. The tryptic peptides were labeled using the iTRAQ 8 Plex Multiplexing kit (Applied Biosystems, Foster City, USA) according to the manufacturer’s protocol. Peptides from different groups were labeled with isobaric tags as follows: reporters 113 for NDC, 117 for LDC, and 115 for HDC, respectively. In order to obtain reliable results, the iTRAQ labeling experiment was replicated with isobaric tags as follows: reporters 114 for NDC, 118 for LDC, and 116 for HDC. After the labeling reactions were performed, the six labeled peptides were mixed, lyophilized, and resuspended in 2% H_3_PO_4_. The peptides were then purified using Strata-X-C (Phenomenex, Torrance, USA) and lyophilized.

#### Nano-high performance liquid chromatography tandem mass spectrometer (Nano-HPLC–MS/MS) analysis

The desalted peptide mixture was delivered in duplicate onto a Acclaim PePmap C_18_-reversed phase column (75 μm × 2 cm, 3 μm, Thermo Scientific, California, USA) and separated with reversed phase C_18_ column (75 μm × 10 cm, 5 μm, Agela Technologies, Wilmington, Delaware, USA) mounted in a nano-HPLC system (SHIMADZU, Kyoto, Japan). Peptides were eluted using a gradient of 5–80% (v/v) acetonitrile in 0.1% formic acid (FA) over 45 min at a flow rate of 300 nL/min combined with a Q Exactive mass spectrometer (Thermo Fisher Scientific, Waltham, MA, USA).

The eluates were directly entered Q-Exactive MS, setting in positive ion mode and data-dependent manner with full MS scan from 350 to 2000 *m/z*, full scan resolution at 70,000, MS/MS scan resolution at 17,500 with minimum signal threshold 1E + 5, isolation width at 2 Da. To evaluate the performance of this mass spectrometry on the iTRAQ labeled samples, two MS/MS acquisition modes, higher collision energy dissociation (HCD) was employed. And to optimize the MS/MS acquisition efficiency of HCD, normalized collision energy (NCE) was systemically examined 28, stepped 20%. Analysis was carried out with 3 technical replications.

#### Data analysis

The raw data was processed using Proteome Discoverer version 1.3 (Thermo Scientific, San Jose, California, USA). For database searching, MS/MS spectra were analyzed using the Mascot algorithm (Version 2.3.0, Matrix Science, Boston, MA, USA) against the Uniprot human sequence database searched with Sequest engine against using the following parameters: full trypsin digest with maximum 1 missed cleavages, fixed modification carbamidomethylation of cysteine (+ 57.021 Da), variable modification Gln ⟶ pyro-Glu of the N-terminus, oxidation of methionine (+ 15.995 Da), and iTRAQ 8-plex modification of lysine and peptide N termini (+ 304.205 Da). We used PeptideProphet and ProteinProphet probabilities ≥ 0.95 to ensure an overall false discovery rate (FDR) below 1% and at least 1 unique peptide was qualified for further quantitative analysis. The fold changes in protein abundance were defined as the median ratios of all spectra significantly matched to the protein with reporter signals between two samples. Proteins with quantification P value < 0.05 in at least two groups and with the ratio > 1.2 (the average ratio of two repeat experiments) were considered as differentially expressed proteins.

### Bioinformatics analysis of differentially expressed proteins

Differentially expressed proteins in HDC vs NDC, LDC vs NDC, and HDC vs LDC groups were further analyzed with the following methods: (A) the gene ontology database (http://www.geneontology.org) was used for the gene ontology (GO) analysis, which was based on three categories: biological process, cellular component and molecular function. P value less than 0.05 using Fisher’s exact test was considered statistically significant. (B) Kyoto Encyclopedia of Genes and Genomes (KEGG) database (http://www.genome.jp/kegg/pathway.html) was used to identify enriched pathways. A two-tailed Fisher’s exact test was conducted to detect for enrichment of proteins in a specific category and pathway enrichment analyses, which tested the enrichment of the differentially expressed proteins against all identified proteins. (C) Hierarchical clustering (HCL) analysis was performed using Cluster 3.0 and Tree View software (http://rana.lbl.gov/EisenSoftware.htm). Based on the tree algorithm, the differentially expressed proteins were organized according to the similarities in the expression profile. (D) The protein–protein interaction (PPI) networks were constructed using STRING online database (http://string-db.org) [31].

### Multiple reaction monitoring (MRM) analysis

#### MRM validation of differentially expressed proteins from iTRAQ

In this project, we developed an MRM method for a total of 53 target proteins. MRM assays were performed according to an experimental procedure described by Zhang et al. [32] with some modifications. Briefly, the peptides with significant MS/MS signals were identified to be used as target peptides in the following MRM experiment using a TripleTOF 6600 MS (AB SCIEX, Concord, USA) equipped with a nano-LC system (SHIMADZU, Kyoto, Japan) to verify whether the peptide had a co-elution chromatogram and the correct retention time. ProteinPilot (AB SCIEX, Framingham, USA) was applied to search against the Uniprot human sequence database, and the MRM transition list was established using Skyline v2.1 (MacCoss Lab, University of Washington, Seattle, USA) [33]. The selected transitions were then adopted to survey the salivary proteins from different groups.

Saliva MRM assays were carried out using a QTRAP 6500 MS (AB SCIEX, Framingham, USA) equipped with an ekspert nanoLC 425 system (AB SCIEX, Framingham, USA). The mobile phases consisted of solvent A (2% acetonitrile with 0.1% FA) and solvent B (98% acetonitrile with 0.1% FA). The peptides were separated using an Eksigent column (75 μm × 15 cm, 3 μm, AB SCIEX, Framingham, USA) at 300 nL/min with a gradient of 5–40% solvent B for 30 min and 40–80% solvent B for 15 min. The MS parameters were set as follows: ionspray voltage at 2300 V, curtain gas at 35.0, ion source gas 1 at 15.0, ion source gas 2 at 0.0, collision gas at high, interface heater temperature at 150.0 °C, entrance potential at 10.0, and Q1 and Q3 at unit resolution.

#### MRM data processing

For MRM analysis, all raw files were imported and processed using Skyline v2.1. MRM peak integrations were manually inspected to ensure correct peak detection, absence of interferences, and accurate integration. MRM signal was defined as the detection of all the transitions from the endogenous peptide exactly coeluting with all the transitions from the stable isotope-labeled peptide. Specificity was confirmed by equivalent retention time and relative areas of light and heavy transitions, while precision was determined by % coefficient of variation (CV, standard deviation divided by the mean).

### Statistical methods

Statistical comparisons processed by SPSS software version 12.0 (Chicago, IL, USA) among three comparison groups were made using unpaired Student’s *t*-tests. P value less than 0.05 was considered statistically significant.

## Results

### iTRAQ-based mass spectrometry profiling of differentially expressed salivary proteins related to dental caries

The caries state of all subjects enrolled in the study was presented in Table 1. Statistical analysis failed to reveal significant differences in age and gender among different groups (P > 0.05). The mean DMFT/dmft for the total group was 3.07, with no significant difference between males and females (P > 0.05). While the mean DMFT/dmft for the LDC and HDC groups was 2.30 and 6.90, respectively, and the difference between the two groups was significant (P < 0.001).

**Table 1:** Caries state of study subjects

Group/gender	Number of subjects	Male/female	DMFT/dmft (mean ± SD)
NDC	10	5/5	0
LDC	10	6/4	2.30 ± 0.67
HDC	10	5/5	6.90 ± 0.99
Male	16	–	2.88 ± 2.83
Female	14	–	3.29 ± 3.27
Total	30	–	3.07 ± 2.99

**Fig. 1:**
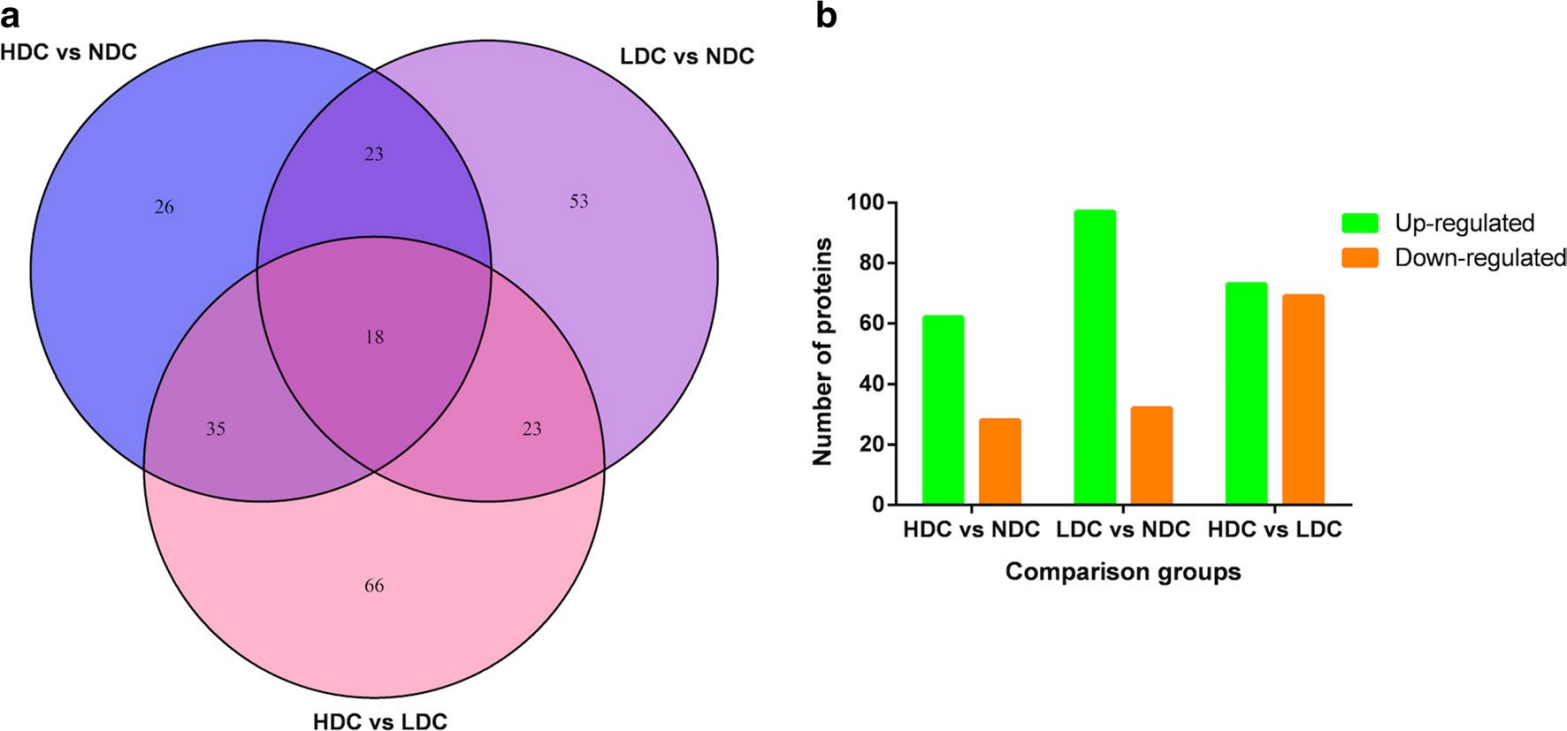
Comparison of differentially expressed proteins identified by iTRAQ in HDC vs NDC, LDC vs NDC and HDC vs LDC groups. **a** Venn diagram for the number of differentially expressed proteins identified commonly or exclusively among three comparison groups. **b** The number of up-regulated proteins and down-regulated proteins in each comparison group

To diminish the influences of between-individual variations, the salivary protein samples from individuals in NDC, LDC and HDC groups were equally pooled respectively (100 μg for each group). The three pooled samples each with a duplicate were iTRAQ-labeled and then analyzed by HPLC–MS/MS. By querying the Uniprot human sequence database with the Mascot algorithm, at 1% FDR both in peptide and protein levels, 36,876 spectrums were matched from 322,562 spectrums. A total of 4369 unique peptides and 759 proteins were identified, and the detailed information regarding the protein identification was listed in Additional file 1. Pearson correlation analysis exhibited good reproducibility and acceptable stability between each experimental group and its replicate (Additional file 2: Fig. S1). By a ratio-fold change > 1.2 and P value < 0.05, 244 proteins were found to be differentially expressed by iTRAQ proteomics. Among them, 18 proteins were commonly present in all comparisons, whereas 26, 53 and 66 differentially expressed proteins were uniquely detected in the comparison groups of HDC vs NDC, LDC vs NDC, and HDC vs LDC, respectively (Fig. 1a and Additional file 3). As compared with NDC group, 62 up-regulated proteins and 28 down-regulated proteins were found in HDC group, while 97 increased proteins and 32 decreased proteins were detected in LDC group (Fig. 1b). Meanwhile, a total of 73 salivary proteins were increased in HDC group relative to LDC group. The remaining 69 decreased proteins in HDC group were up-regulated in LDC group (Fig. 1b). The comparison of the log ratio of relative intensity for differentially expressed proteins identified in three groups was illustrated in Additional file 2: Fig. S2.

### Functional classification of differentially expressed proteins among different comparison groups

**Fig. 2:**
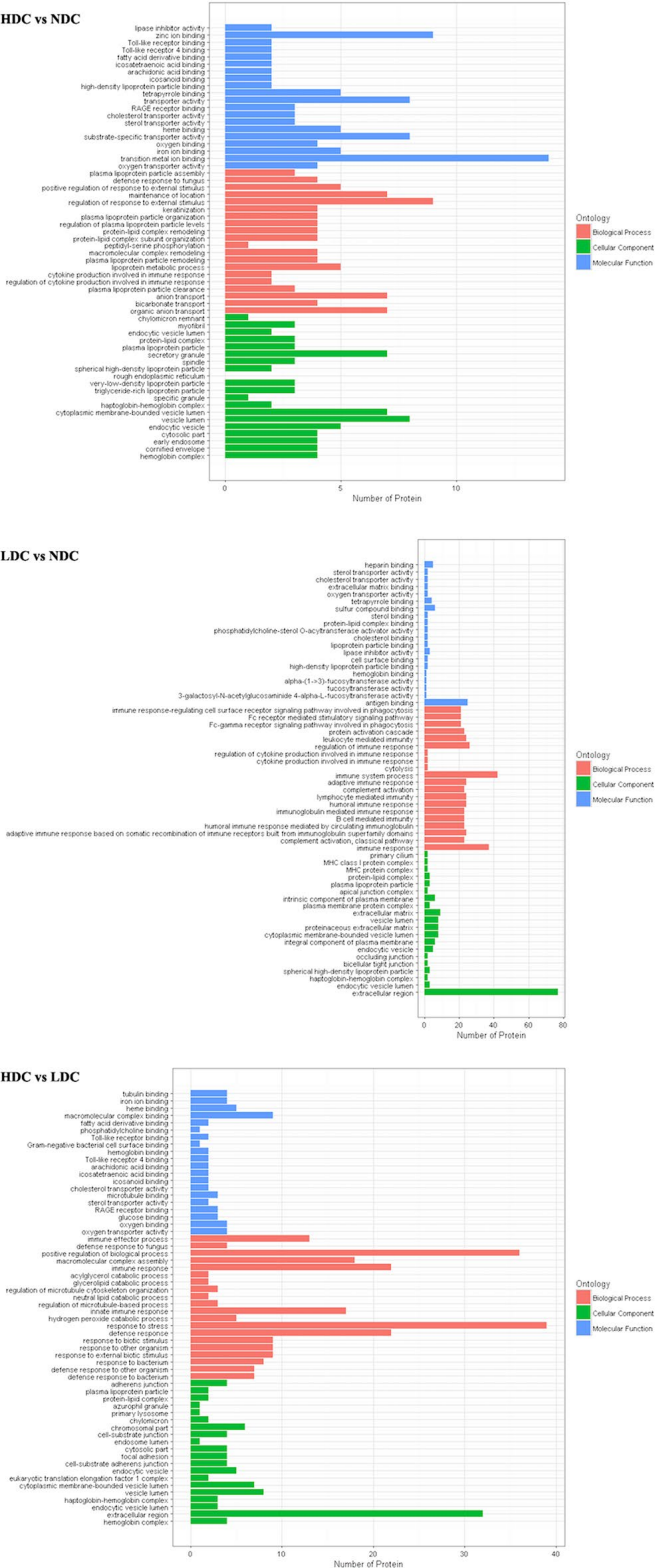
Up-regulated proteins were functionally annotated for biological process, cellular component and molecular function in HDC vs NDC, LDC vs NDC, and HDC vs LDC groups

To further study the biological function of the differentially expressed proteins, they were cataloged by the GO analysis according to biological processes, molecular function, and cellular components. In comparison with healthy controls, GO analysis showed that proteins involved in transition metal ion binding (24.1%) were enriched in up-regulated proteins in HDC group, whereas proteins in extracellular space (21.5%) and involved in immune system process (17.2%) were enriched in up-regulated proteins in LDC group (Fig. 2). On the other hand, proteins involved in response to stress (12.7%) and positive regulation of biological process (13%) were enriched in up-regulated proteins in HDC group compared with LDC group (Fig. 2). As for the down-regulated proteins among different comparison groups, the number of proteins assigned in three different classifications was exhibited in Additional file 2: Fig. S3.

**Fig. 3:**
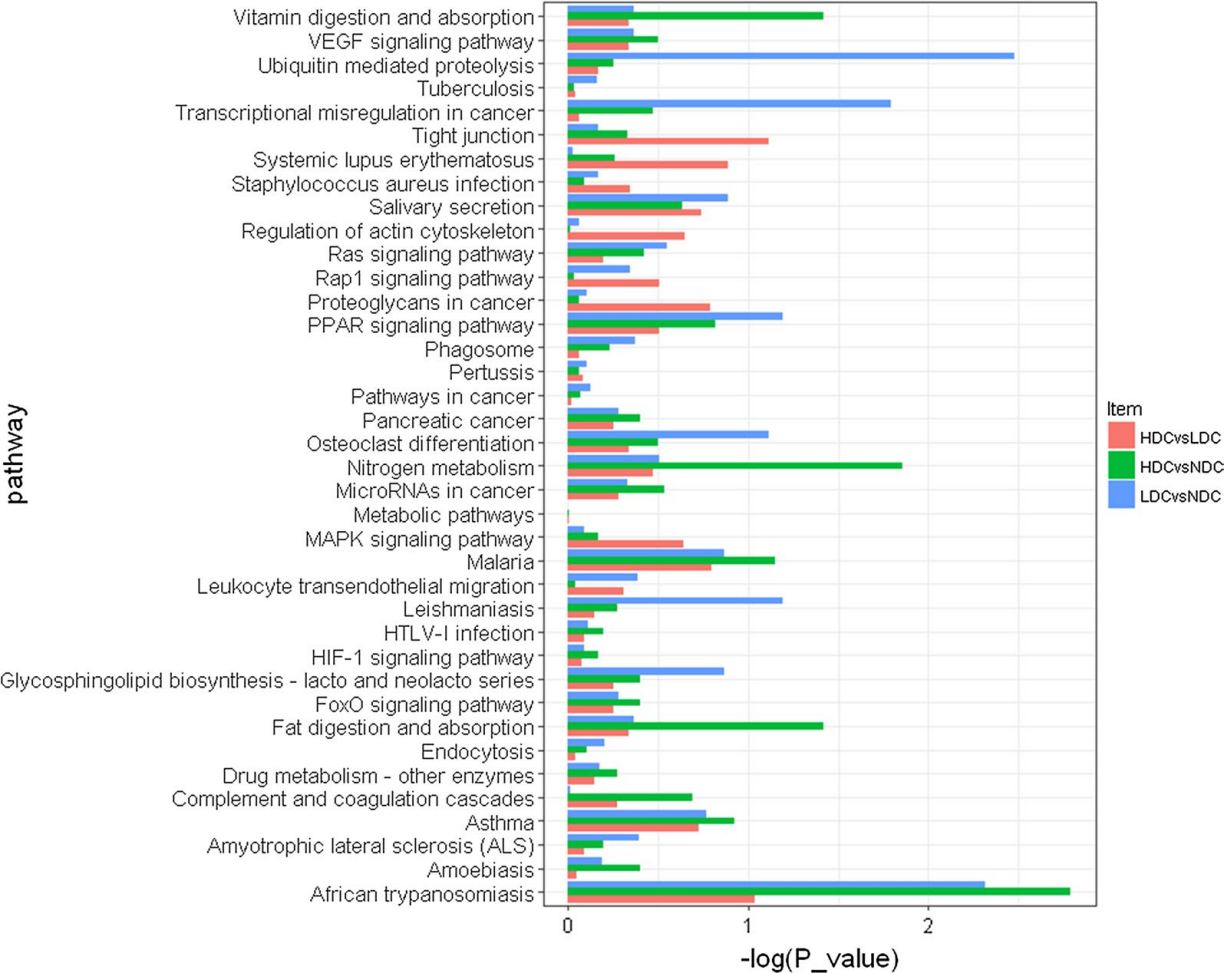
Pathway analysis of differentially expressed proteins found commonly in HDC vs NDC (green), LDC vs NDC (blue), and HDC vs LDC (red) groups

The differentially expressed proteins were then analyzed using KEGG pathways database. As compared with NDC, the differentially expressed proteins were classified into 61 pathways, 27 and 25 of which were respectively only involved by the up-regulated and down-regulated proteins in HDC (Additional file 2: Fig. S4, Additional file 4: 4-1). In the comparison LDC vs NDC, the significantly enriched pathways consisted of the ubiquitin mediated proteolysis pathway and salivary secretion pathway (Additional file 4: 4-2). In addition, the top pathway involved by the up-regulated proteins in HDC relative to LDC was the metabolic pathway, followed by regulation of actin cytoskeleton pathway and focal adhesion pathway (Additional file 2: Fig. S4, Additional file 4: 4-3). Moreover, the up-regulated proteins in LDC were enriched in the salivary secretion pathway, when this group was compared with NDC and HDC respectively. On the other hand, 38 pathways were commonly detected in all three comparison groups, in which the differences were more marked between healthy children and children with dental caries, particularly in the ubiquitin mediated proteolysis pathway and African trypanosomiasis pathway (Fig. 3).

Next, to reveal the representative feature of childhood caries salivary proteome, we attempted to distinguish the differentially expressed proteins in different groups by using hierarchical clustering (HCL) analysis. HCL analysis revealed the differential expression trend of the significant proteins in NDC, LDC and HDC, indicating that the present strategy indeed added to discover salivary proteins, which were effective to distinguish patients with varying degree of dental caries from caries-free individuals. In addition, HCL was also conducted to analyze the experimental groups and their replicates, nicely separating the three sample groups (Additional file 2: Fig. S5).

**Fig. 4:**
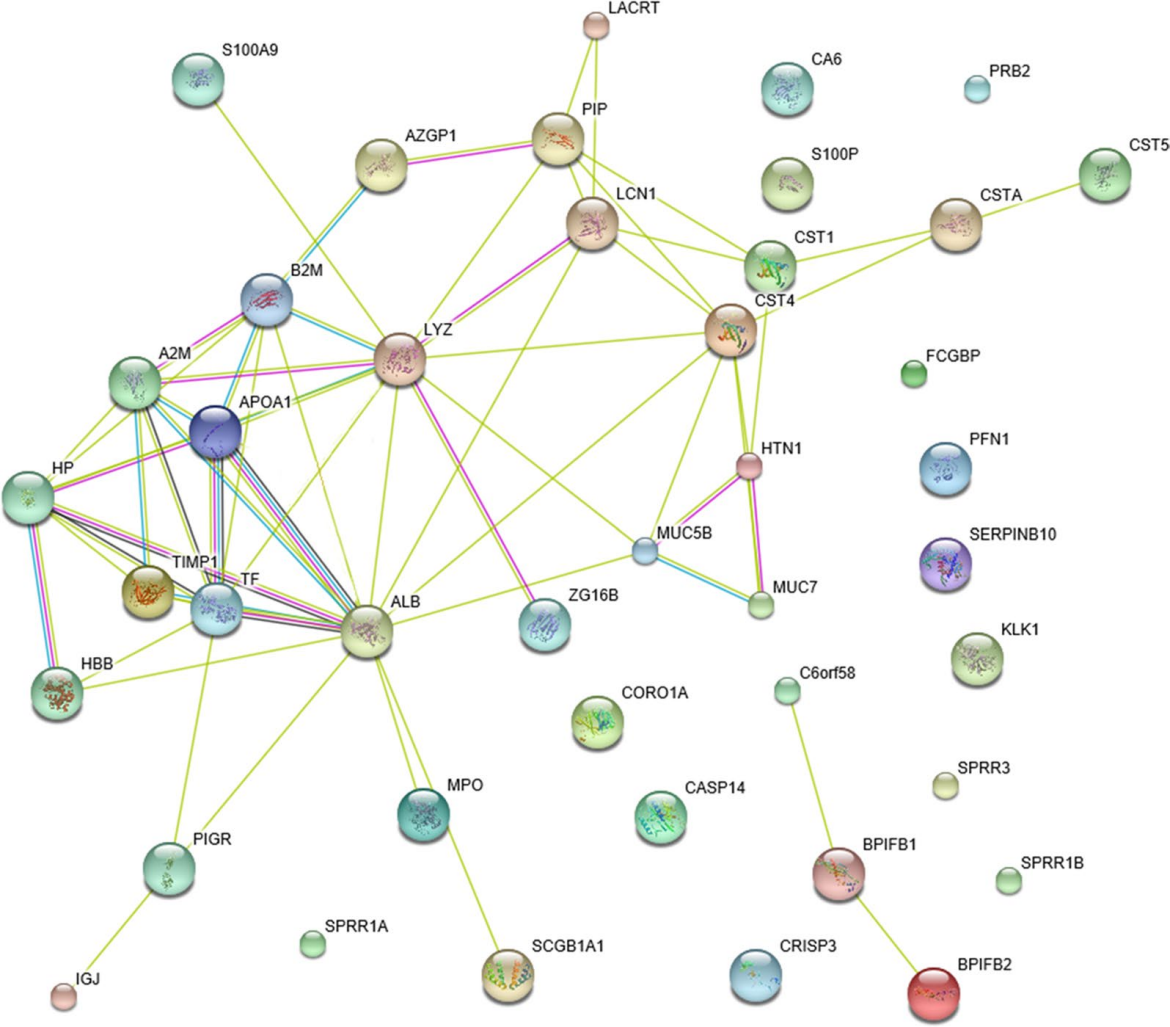
Functional interaction networks of target proteins validated by MRM. The protein–protein interaction networks consisting of 42 proteins were listed in Additional file 4: Table S4, where depicted 63 interaction links between individual nodes/proteins. The light blue lines represent database evidence; the purple lines represent experimental evidence; the yellow lines represent text mining evidence; and the black lines represent co-expression evidence

Moreover, to highlight the biological processes potentially involved in caries resistance or cariogenicity in saliva, the protein–protein interaction (PPI) network of proteins differentially expressed in three comparison groups with successful validation were conducted using STRING online database, excluding 11 proteins without information in STRING database. A total of 63 interaction links between proteins were depicted in the built PPI network (Fig. 4, Additional file 5), in which two separate interaction networks were predicted. Lysozyme was a key protein and interacted with 11 proteins in this network, including cystatin S, mucin 5B and protein S100 A9. Additionally, the module constituted by cystatin S, mucin 5B, mucin 7 and histatin 1 was displayed here, in which these four proteins interacted with each other. And the other separated network showed the interaction of BPI fold containing family B member 1 with BPI fold containing family B member 2 and chromosome 6 open reading frame 58.

### Targeted quantitation of candidate proteins using MRM-MS

**Fig. 5:**
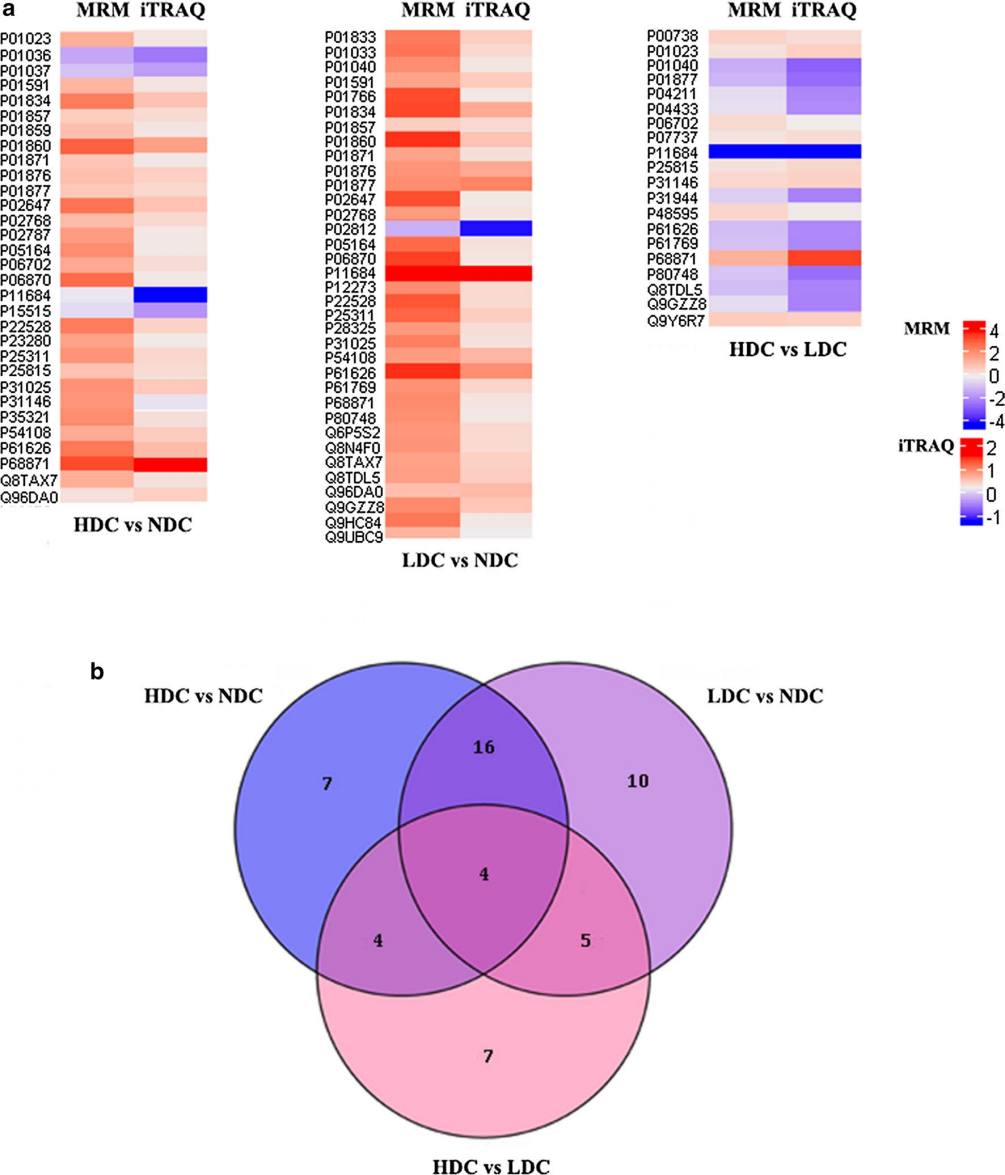
Verification of target proteins using MRM assay. **a** Heat map showing the change in abundance of differentially expressed proteins in HDC vs NDC, LDC vs NDC, and HDC vs LDC groups, as measured using MRM (left panel) and iTRAQ (right panel). The protein names corresponding to the accession according to Uniprot database were listed in Additional file 4: Table S5. **b** Venn diagram for the number of proteins verified by MRM commonly or exclusively among three comparison groups

Using the established MRM assay, the differentially expressed proteins identified by iTRAQ were further verified. In the present study, MRM analysis succeeded in validating 31, 35 and 20 proteins in comparison groups of HDC vs NDC, LDC vs NDC, and HDC vs LDC, respectively. The changes observed in the abundances of these target proteins, quantitatively measured using MRM and iTRAQ, were then compared. The detailed information from MRM and iTRAQ analysis of three comparison groups was supplied in Additional file 6: 6-1. As a result, all these target proteins showed the same trend of changes in abundance in the three comparisons between MRM and iTRAQ, respectively (Fig. 5a).

Among the validated proteins, 4 proteins were shared in all three comparisons, such as lysozyme C, whereas 7, 10 and 7 target proteins were specifically detected in comparison of HDC vs NDC, LDC vs NDC, and HDC vs LDC, respectively (Fig. 5b, Additional file 6: 6-2). Of the seven unique proteins in comparison between HDC and NDC, four up-regulated proteins (carbonic anhydrase 6, serotransferrin, cornifin A and Ig gamma-2 chain C region) and three down-regulated proteins (cystatin S, cystatin SN and histatin 1) in HDC from iTRAQ data had coincident expression with these from MRM analysis. Four proteins shared in HDC vs NDC and HDC vs LDC, including protein S100 A9, protein S100 P, alpha 2 macroglobulin and coronin 1A, were all up-regulated in HDC group. For the specific differentially expressed proteins in HDC vs LDC, four significantly up-regulated proteins (haptoglobin, profilin-1, serpin B10, IgGFc-binding protein) and three significantly down-regulated proteins (Ig lambda chain V region 4A, Ig kappa chain V-III region VG, caspase-14) in HDC from iTRAQ data were also showed up-regulated and down-regulated respectively in MRM analysis. Besides, there were 16 proteins commonly present in two comparison groups of HDC vs NDC, LDC vs NDC, all of which, including mucin 7, were significantly up-regulated in both HDC and LDC compared with NDC. And these shared up-regulated proteins in two disease groups were identified to be associated with the biological process of immune response and immune system process.

## Discussion

Paediatric dental caries is considered to be a major public health problem. As an endogenous factor, saliva and its components responsible for playing important roles in protecting oral structures, may partially determine why some children develop caries whereas others do not. The purpose of the present study was to explore significant salivary proteins involved in anti-cariogenicity or cariogenicity through comparing proteome profiling of saliva from children with dental caries to that of orally healthy individuals, as we hypothesized that the components of saliva might be associated with caries status. Proteomics and related techniques have advanced significantly over the last two decades, making this analysis possible. However, due to the lack of a coherent, demonstrably successful pipeline from the discovery to the verification stage, previous reports regarding to the proteome profiling of dental caries remained controversial. Herein, our study revealed the salivary proteome of the children with and without dental caries using iTRAQ-based MRM-MS for quantitative proteomic analysis.

In this study, 4369 unique peptides and 759 proteins were identified, which are much more than that in salivary peptidome and proteome profiling of childhood dental caries in recent studies [18, 34]. The higher number of salivary protein identification in our study is probably due to the differences in sampling methods, grouping methods, saliva-based proteomic approaches and the subjects included in studies. Considering that stimulated saliva samples could be diluted the concentration of proteins, unstimulated saliva samples used in our study may be preferred for in-depth analysis of the salivary proteome. In order to avoid false identification of biomarkers due to nondisease related differences between children with and without dental caries, we controlled the inter-individual differences within groups and enrolled subjects with similar mean age and demographic characteristics to the diseased groups. Also as saliva is susceptible to many physiological and biochemical processes, all the saliva specimens were collected and processed consistently according to the strict method described above. Thus, the quality of samples and consequently the resulting data are reliable.

Since dental caries is a progressive disease, there is an important role for high-throughput methods to characterize the proteins involved in the disease process. Therefore, we divided the subjects into three groups according to the disease severity based on DMFT/dmft index. We postulated that some proteins in saliva of children with caries might be associated with the severity of disease. Consequently, we found 142 proteins expressed differentially in HDC vs LDC, of which 66 proteins were exclusive in this comparison from the results of iTRAQ. Besides, 40 of the 66 unique proteins were up-regulated in HDC, indicating their potential roles in the exacerbation of childhood dental caries. The up-regulated proteins involved in the response to stress and positive regulation of biological process in terms of GO annotation were enriched in HDC group compared with LDC group, and they were also categorized into 80 pathways, which were most represented by these proteins in metabolic pathways, focal adhesion as well as regulation of actin cytoskeleton. Among them, the proteins alpha actinin 1 and myosin regulatory light chain 12A both involved in the pathways for focal adhesion and regulation of actin cytoskeleton were associated with cell proliferation and cell moving [35].

As for the differentially expressed proteins in HDC vs NDC, there were 62 up-regulated proteins in HDC groups from iTRAQ results, such as mucin 7, protein S100 A9, alpha-2-macroglobulin, zinc-alpha-2-glycoprotein, zymogen granule protein 16 homolog B and Ig kappa chain C region, which were also successfully validated in MRM analysis. As a result of MRM, protein S100 A9, protein S100 P, coronin 1A and alpha-2-macroglobulin were shared in HDC vs NDC and HDC vs LDC with higher expression levels in HDC group, indicating their potential diagnostic values in the childhood dental caries. In terms of the comparison between LDC and NDC, 97 salivary proteins were up-regulated in LDC, including mucin 7, mucin 5B, Ig kappa chain V-III region, which were also regarded as the special proteins of children with dental caries in a previous study [34]. In addition, from the iTRAQ results, we found that complement C4-B was up-regulated in LDC when compared with NDC. Likewise, it has been reported that active components of the complement system in salivary metaproteome were associated with dental caries [19], but their exact roles in the progression of dental caries still need further research. More importantly, of the unique proteins in LDC vs NDC in MRM analysis, basic salivary proline rich protein 2 was the only one down-regulated protein in LDC. The protective property of basic proline rich proteins (PRPs) in caries prevention has been reported previously, which was found to be able to neutralize acid produced by streptococci through attaching to a major adhesion antigen on the surface of *S. mutans* and other oral streptococci [36].

Both mucin 7 and mucin 5B are implicated in the prevention of dental cavity formation. The importance of mucin 7 in caries prevention has been demonstrated in elderly populations, who with lower mucin 7 concentrations were found to have higher *S. mutans* titers in saliva [37]. In fact, mucin 7 was able to bind to *S. mutans* directly through the bacterium’s alpha-enolase surface protein, while mucin 5B could reduce the attachment and biofilm formation of *S. mutans*, thereby accelerating the clearance of bacteria from the oral cavity [38, 39]. As we know, salivary proteins seldom act alone, and they often bind together to perform their biological functions. It may thus be unrealistic to expect any single salivary factor to be adequate in protecting the integrity of teeth and counteracting the caries process. An important way mucin 7 and mucin 5B protect the oral cavity is by binding to select group of salivary proteins, through which they can influence the proteins’ localization in the oral cavity, increase their retention time, and then alter their biological activity [40]. In this study, PPI network analysis was conducted to embed the differential salivary proteome with a biological framework, in which mucin 7, mucin 5B, histatin 1 and cystatin S interacted with each other. Histatin 1 was found to bind the N-terminal domain on the mucin 7 polypeptide backbone [41], and mucin 5B also formed heterotypinc complexes with the same salivary proteins as mucin 7 [42]. As these proteins all have antimicrobial properties, the complexes formation could increase their availability in saliva, protect proteins from proteolytic degradation and be beneficial to oral health. However, further studies are needed to better understand the effect of this complex on the biological activity of each component.

Among the differentially expressed proteins in different comparisons, the up-regulated proteins in NDC may also provide the source for the anti-cariogenic factors. Through iTRAQ analysis, statherin was found to be significantly up-regulated in NDC with the highest fold change in HDC vs NDC. In addition, among the unique proteins in comparison between HDC and NDC, cystatin S, cystatin SN and histatin 1 were down-regulated in HDC in MRM analysis. As for histatin 1, the value of MRM ratio for which was 0.71 in HDC compared with NDC, was involved in the biological process of biomineralization, antibacterial and antifungal response. Interestingly, these results were in line with previous findings. Vitorino et al. [43] analyzed samples from caries-free and caries-susceptible subjects and revealed a strong correlation between the absence of dental caries and large amounts of histatin 1 and statherin, indicating the importance of these proteins in the maintenance of tooth integrity. Also, their subsequent study found significantly higher quantities of cystatin S and cystatin SN from caries-free group [44]. These phosphorylated proteins, including histatin, statherin and cystatin, which were shown to maintain calcium saturation in saliva around teeth and then promoted the process of remineralization, may play important roles in the inhibition of caries process [7, 45]. Although the differentially expressed salivary proteins in our study might be nonspecific to childhood caries, they seem to demonstrate an abnormal oral condition of those young children susceptible to dental caries. There are still more work to further investigate the mechanism of salivary biomarkers for dental caries in a larger sample size, and translate them from the laboratory level into the clinical practice.

## Conclusions

The present study utilized iTRAQ/MRM methodology to characterize salivary components and their interactions, and constructed the comparative proteomics map for childhood dental caries, which increases knowledge about the salivary proteins functioned in this oral disease. Specifically, some key screened proteins, such as protein S100 A9, mucin 7, mucin 5B, statherin, histatin 1, cystatin S, cystatin SN and basic salivary proline rich protein 2, are worth studying for validation in a larger sample size in future studies. And these differentially expressed proteins in whole saliva with potential anti-cariogenic function in this study can be useful for drawing up caries-preventive agents for individualized preventive strategies in the future.

## Additional files

**Additional file 1.** List of proteins commonly identified by iTRAQ in all saliva samples.

**Additional file 2: Figure S1.** The correlation analysis between each experimental group and its replicate in iTRAQ quantification. **Figure S2.** Log ratio of relative intensity for differentially expressed proteins in HDC vs NDC (A), LDC vs NDC (B) and HDC vs LDC (C) groups. **Figure S3.** Functional annotation of down-regulated proteins for biological process, cellular component and molecular function in HDC vs NDC, LDC vs NDC, and HDC vs LDC groups. **Figure S4.** Pathway analysis of up-regulated and down-regulated proteins based on KEGG in HDC vs NDC (A), LDC vs NDC (B) and HDC vs LDC (C) groups. **Figure S5.** Hierarchical Clustering analysis of differentially expressed proteins found commonly in HDC vs NDC, LDC vs NDC, and HDC vs LDC groups. Saliva samples are shown in the columns, and proteins are shown in the rows.

**Additional file 3.** Category list of differentially expressed proteins commonly or uniquely detected in HDC vs NDC, LDC vs NDC, and HDC vs LDC.

**Additional file 4.** Category list of pathways involved by differentially expressed proteins in HDC vs NDC (1), LDC vs NDC (2), and HDC vs LDC (3).

**Additional file 5.** Detailed information from PPI analysis of 42 target proteins.

**Additional file 6.** Relative protein abundances for 53 target proteins (1), and category list of these proteins commonly or uniquely detected in HDC vs NDC, LDC vs NDC, and HDC vs LDC using MRM (2).

## Abbreviations

iTRAQ: isobaric tags for relative and absolute quantitation
MRM: multiple reaction monitoring
NDC: no dental caries
LDC: low dental caries
HDC: high dental caries
MMP-8: matrix metalloproteinase-8
HEPES: 4-(2-hydroxyethyl)-1-piperazineethanesulfonic acid
PMSF: phenylmethanesulfonyl fluoride
EDTA: ethylene diamine tetraacetic acid
DTT: dithiothreitol
BSA: bovine serum albumin
FA: formic acid
HCD: higher collision energy dissociation
MS: mass spectrometer
Nano-HPLC–MS/MS: nano-high performance liquid chromatography tandem mass spectrometer
FDR: false discovery rate
GO: gene ontology
KEGG: kyoto encyclopedia of genes and genomes
HCL: hierarchical clustering
PPI: protein–protein interaction
CV: coefficient of variation
PRPs: proline rich proteins
