# Supplementary material for: Comparative salivary proteomics analysis of children with and without dental caries using the iTRAQ/MRM approach
Source: J Transl Med. 2018 Jan 19;16:11. doi: 10.1186/s12967-018-1388-8 (PMC5775567; doi:10.1186/s12967-018-1388-8)

Comparative salivary proteomics analysis of children with and without dental caries using the iTRAQ/MRM approach.

Kun Wang, Yufei Wang, Xiuqing Wang, Qian Ren, Sili Han, Longjiang Ding, Zhongcheng Li, Xuedong Zhou, Wei Li, Linglin Zhang*

State Key Laboratory of Oral Diseases & National Clinical Research Center for Oral Diseases & Dept. of Cariology and Endodontics West China Hospital of Stomatology, Sichuan University, Chengdu, Sichuan, China.

*Corresponding author: Linglin Zhang, Professor, State Key Laboratory of Oral Diseases & National Clinical Research Centre for Oral Disease, Dept. of Cariology and Endodontics West China Hospital of Stomatology, Sichuan University, Chengdu, China. Postal Address: No.14, Section 3 of Renmin South Road, Chengdu, China. Telephone: 86-028-85503470; E-mail: zhll_sc@163.com.

Fig. S1. The correlation analysis between each experimental group and its replicate in iTRAQ quantification.

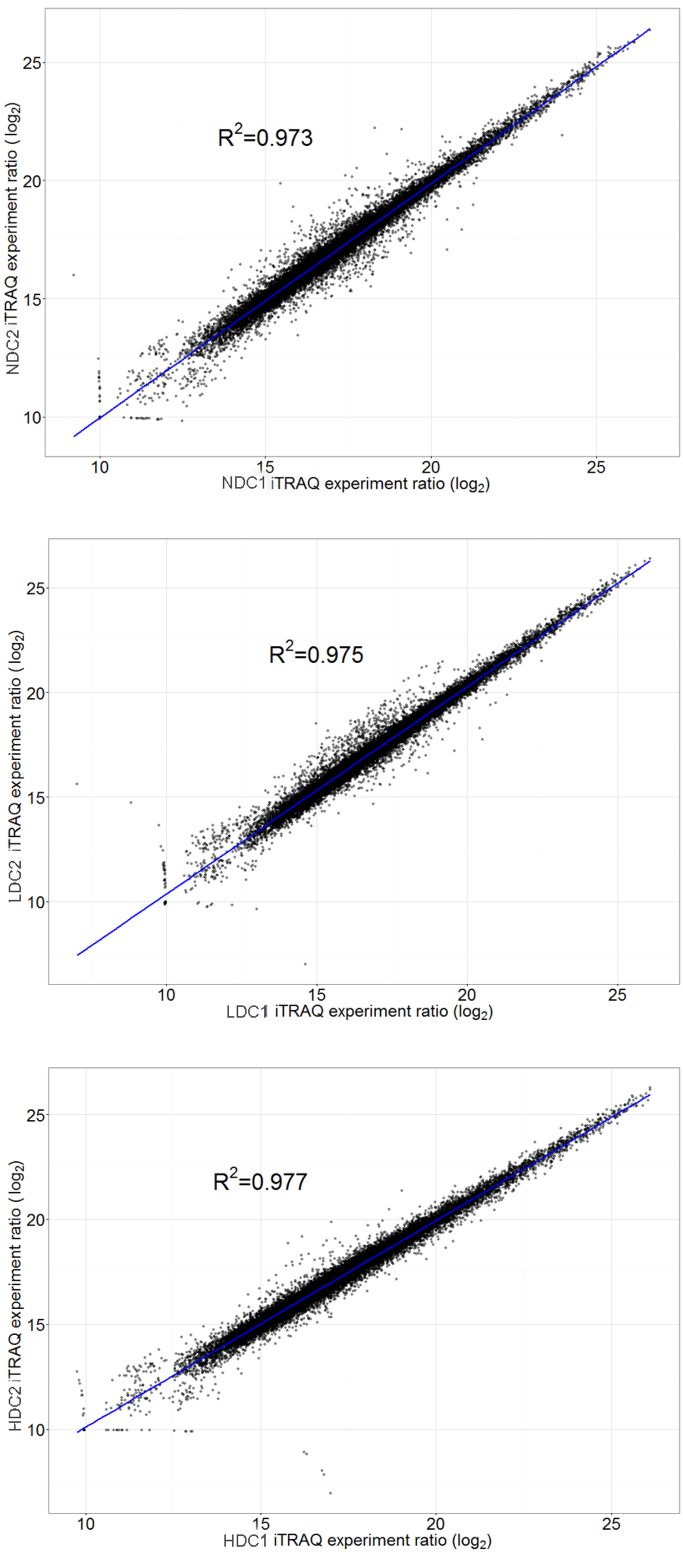

Fig. S2. Log ratio of relative intensity for differentially expressed proteins in HDC vs NDC (A), LDC vs NDC (B) and HDC vs LDC (C) groups.

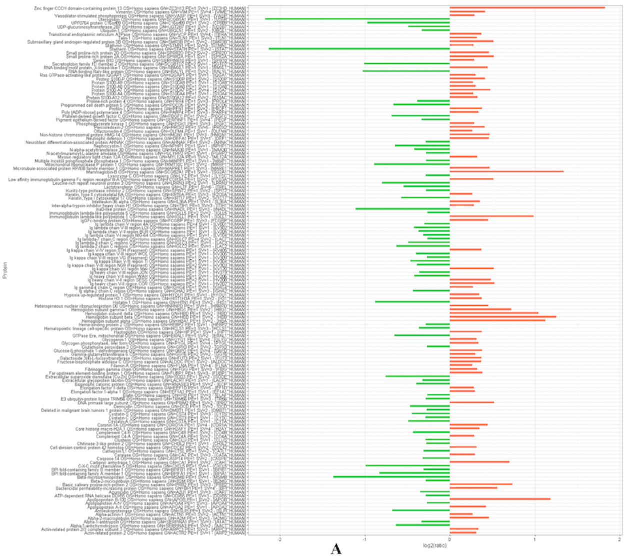

A

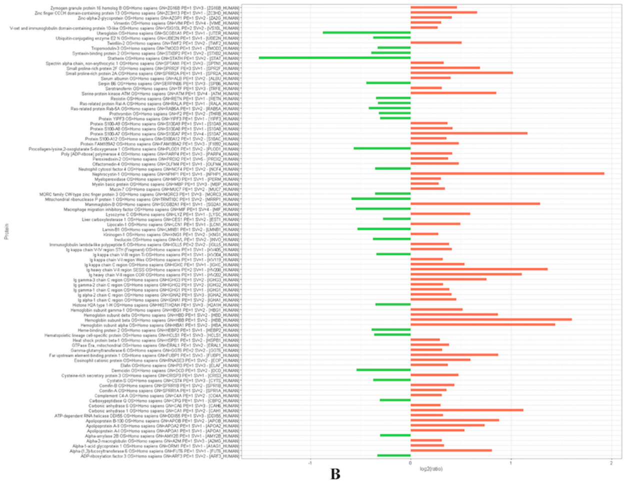

B

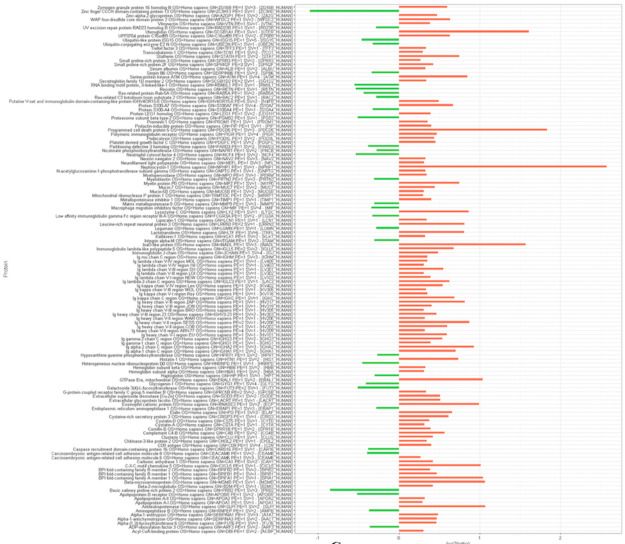

C

Fig. S3. Functional annotation of down-regulated proteins for biological process, cellular component and molecular function in HDC vs NDC, LDC vs NDC, and HDC vs LDC groups.

HDC vs NDC

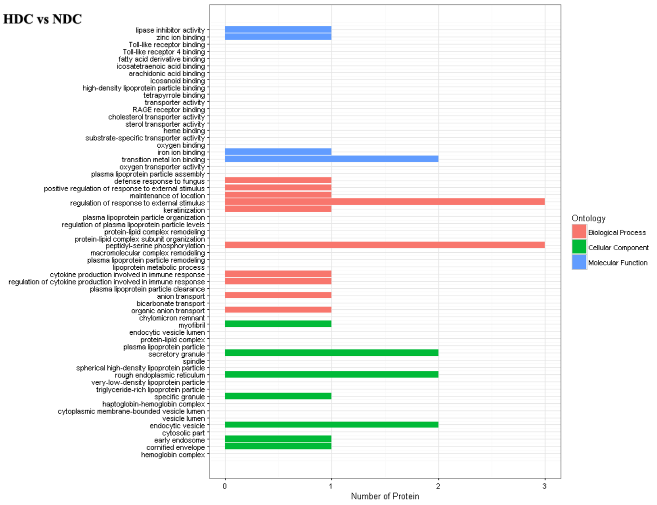

LDC vs NDC

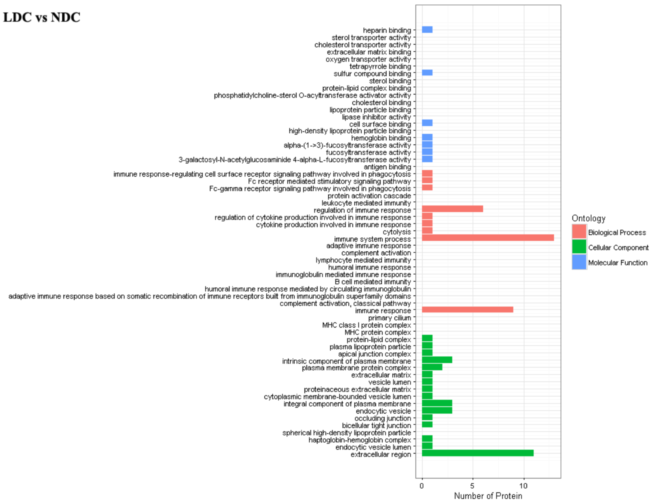

HDC vs LDC

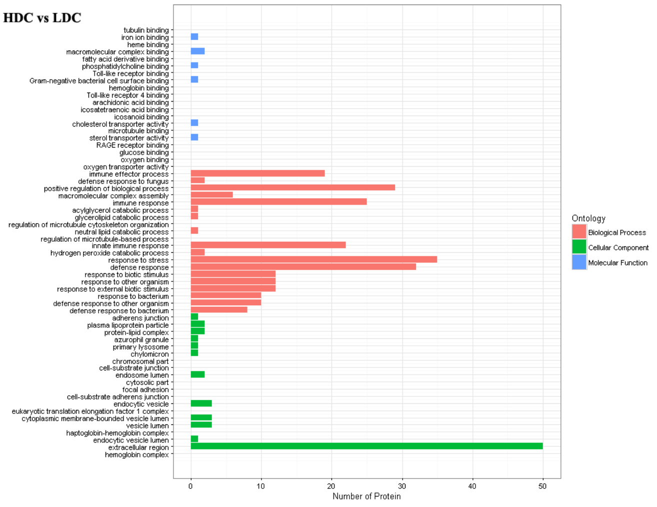

Fig. S4. Pathway analysis of up-regulated and down-regulated proteins based on KEGG in HDC vs NDC (A), LDC vs NDC (B) and HDC vs LDC (C) groups. Differentially expressed proteins were classified in different comparison groups according to the KEGG database.

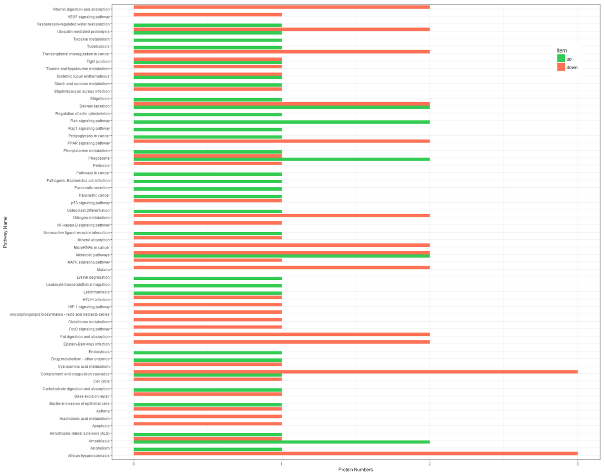

A

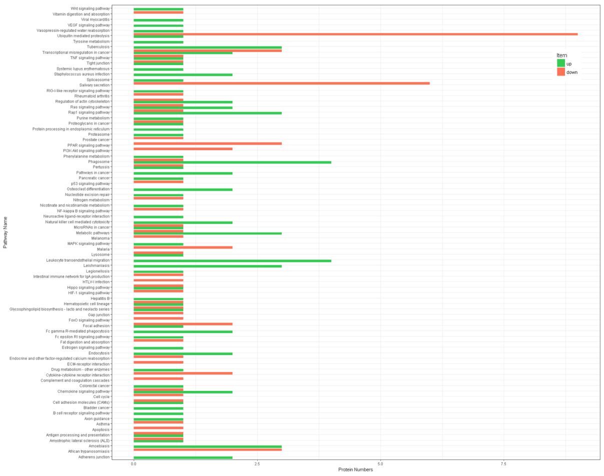

B

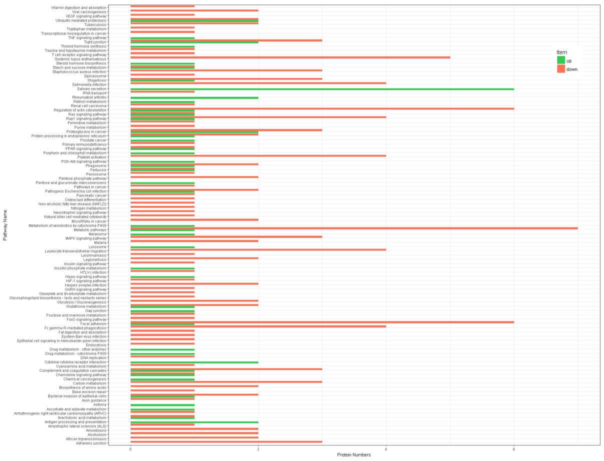

C

Fig. S5. Hierarchical Clustering analysis of differentially expressed proteins found commonly in HDC vs NDC, LDC vs NDC, and HDC vs LDC groups. Saliva samples are shown in the columns, and proteins are shown in the rows.

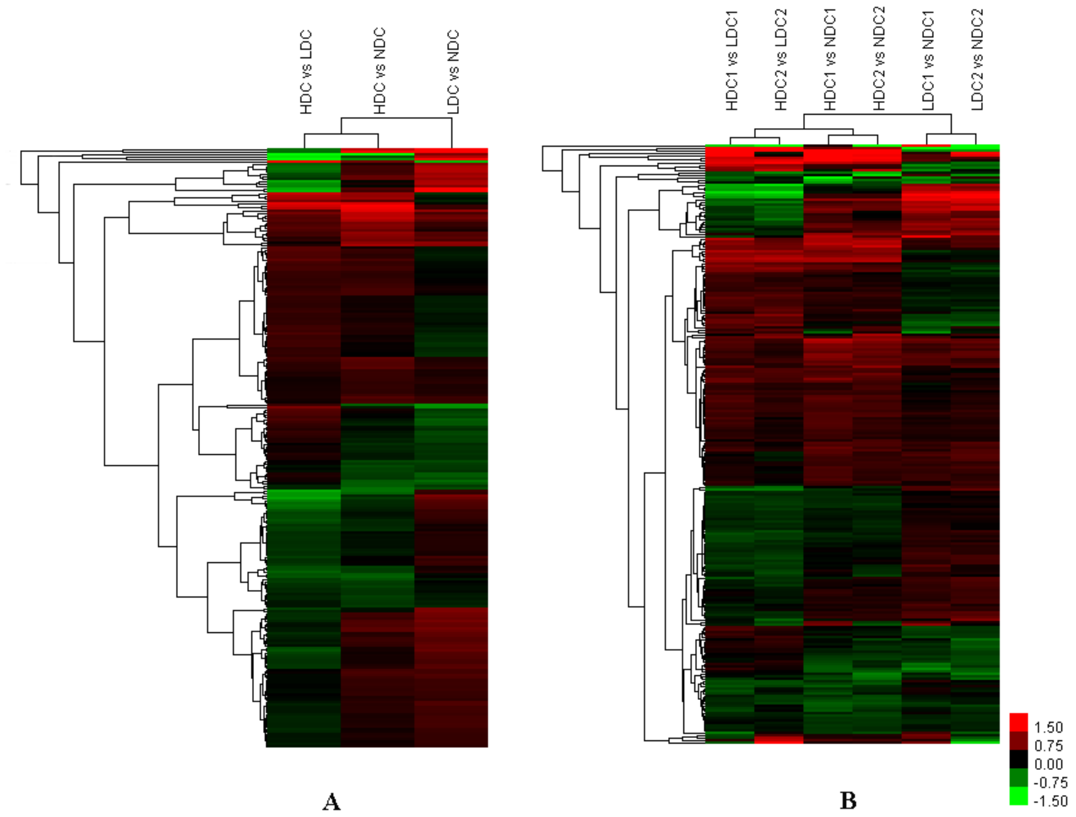

Supplement: Supplementary file 2 — Additional file 2: Figure S1. The correlation analysis between each experimental group and its replicate in iTRAQ quantification. Figure S2. Log ratio of relative intensity for differentially expressed proteins in HDC vs NDC (A), LDC vs NDC (B) and HDC vs LDC (C) groups. Figure S3. Functional annotation of down-regulated proteins for biological process, cellular component and molecular function in HDC vs NDC, LDC vs NDC, and HDC vs LDC groups. Figure S4. Pathway analysis of up-regulated and down-regulated proteins based on KEGG in HDC vs NDC (A), LDC vs NDC (B) and HDC vs LDC (C) groups. Figure S5. Hierarchical Clustering analysis of differentially expressed proteins found commonly in HDC vs NDC, LDC vs NDC, and HDC vs LDC groups. Saliva samples are shown in the columns, and proteins are shown in the rows. [file 12967_2018_1388_MOESM2_ESM.pdf]
